# Non-Linearity in Wide Dynamic Range CMOS Image Sensors Utilizing a Partial Charge Transfer Technique

**DOI:** 10.3390/s91209452

**Published:** 2009-11-26

**Authors:** Suhaidi Shafie, Shoji Kawahito, Izhal Abdul Halin, Wan Zuha Wan Hasan

**Affiliations:** 1 Department of Electrical and Electronic Engineering, Faculty of Engineering, Universiti Putra Malaysia, 43400 UPM Serdang, Selangor, Malaysia; E-Mails: izhal@eng.upm.edu.my (I.A.H.); wanz@eng.upm.edu.my (W.Z.W.H.); 2 Research Institute of Electronics, Shizuoka University, 3-5-1 Johoku, Nakaku, Hamamatsu 432-8011, Japan; E-Mail: kawahito@idl.rie.shizuoka.ac.jp

**Keywords:** electronic imaging, CMOS image sensor, wide dynamic range, partial charge transfer, non-linearity

## Abstract

The partial charge transfer technique can expand the dynamic range of a CMOS image sensor by synthesizing two types of signal, namely the long and short accumulation time signals. However the short accumulation time signal obtained from partial transfer operation suffers of non-linearity with respect to the incident light. In this paper, an analysis of the non-linearity in partial charge transfer technique has been carried, and the relationship between dynamic range and the non-linearity is studied. The results show that the non-linearity is caused by two factors, namely the current diffusion, which has an exponential relation with the potential barrier, and the initial condition of photodiodes in which it shows that the error in the high illumination region increases as the ratio of the long to the short accumulation time raises. Moreover, the increment of the saturation level of photodiodes also increases the error in the high illumination region.

## 1. Introduction

Due to their ability to automatically produce clear images of an object plane that has extremely varying illumination levels, wide dynamic range image sensors are required for many applications such as cameras for security systems, automobiles and industry. There have been various approaches to enhance the dynamic range of CMOS image sensors [1-6]. However, the non-linear response type wide dynamic range CMOS image sensor are not preferable for color images [1,2], while the linear response type such as CMOS image sensor with in-pixel lateral overflow integration capacitor is not appropriate for small pixels due to their complex pixel structure [3]. The multiple exposures technique is one of the solutions for dynamic range enhancement. However the conventional technique of multiple exposures has a problem with the motion artifact due to signal loss during integration [4-6]. One of the most recent techniques to enhance the dynamic range of CMOS image sensors is the partial charge transfer technique [7,8]. It provides a signal at short accumulation in addition to a signal at long accumulation by continuing charge accumulation. In this technique, there is no degradation of the sensitivity in the wide dynamic range operation [7]. The difference of two charge accumulation time in one frame period expands the dynamic range of the sensor. This technique uses a normal four transistors active pixel sensor (4T-APS) with an adjustable midpoint drive for the transfer gate. By using two types of accumulation time and two different drives to control the transfer gate during accumulation and readout, two set of output signals can be obtained from a single photodiode. The wide dynamic range signal can be synthesized from the two types of signals mentioned. Although it enhances the dynamic range of the sensor, the partial charge transfer technique also contributes to less sensitivity loss as compared to the conventional dynamic range expansion technique because it makes efficient use of full exposure by continuation of charge integration and maximize the fill factor. However, the short accumulation signal obtained from partial transfer operation is non-linear with respect to the incident light [9]. In this paper, an analysis of the nonlinearity due to the partial charge transfer in the synthesized signals has been carried, and the relationship between the dynamic range and the non-linearity is deliberated by discussing out the pixel structure, the dynamic range expansion by partial charge transfer, the principle of charge transfer, the non-linearity due to current diffusion and the non-linearity due to initial condition of photodiode.

## 2. Pixel Structure

**Figure 1.**
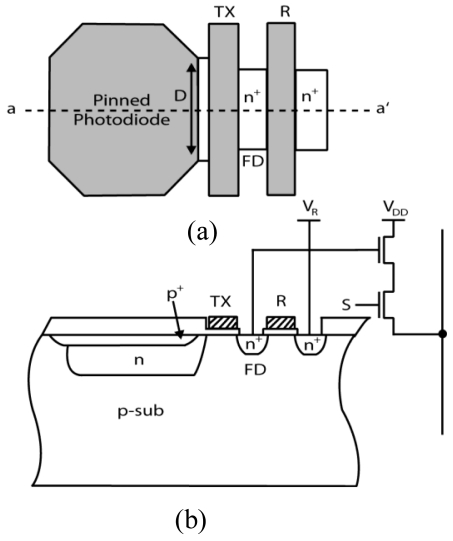
**(a)** Simplified layout and **(b)** cross section at line aa', of the pixel.

The simplified layout of the pixel and its cross-section at line aa' are shown in Figures 1a,b, respectively. The pixel is a normal 4T-APS. However, the supply voltage for the transfer gate driver is designed to be adjustable for partial and whole charge transfer. To achieve high performance, the photodiode in the pixel has to be optimized. The shape of photodiode layout, the structure of photodiode, and the layout have significant influences on the performance of the whole imager [10,11]. In the pixel, the photodiode (PD) is designed with an octagonal shape with a small width, *D* that is placed on the PD side for the purpose of improving the imager's performance. The use of buried PD is aimed to reduce the dark current of the pixel. However, the n layer near the transfer gate is not totally covered by p^+^ layer and still contributes to the dark current. Therefore, by using a small *D*, the dark current can be reduced because the area of uncovered n layer is decreased.

The use of octagonal shape for PD can increase the speed of charge flow during charge transfer operation, preventing image lag [12]. In rectangular PD, the accumulated charge remains at the corners of PD during charge transfer operation resulting in a slower charge transfer. Moreover, the octagonal shape of photodiode dedicated to 12% lower interconnection surface and has a better spectral response compared to the rectangular photodiode [13]. Besides, using the normal 4T-APS can be an advantage for this technique because high field factor can easily be obtained. Table 1 shows the characteristics of the simulated pixel.

## 3. Dynamic Range Expansion by Partial Charge Transfer

The principle of the dynamic range expansion by the partial charge transfer technique is discussed in this section. In this sensor, one frame of accumulation time is divided into two sub-frames.

The next steps explain the operation of the wide dynamic range image sensor with partial transfer:

1. When a strong light is irradiated on the pixel, the accumulated charge in the PD reaches the saturation level (*Q_max_*) in short time. Since the signal is saturated, it cannot be read out at this time, therefore, the accumulated charge is partially drained and charge accumulation is repeated. The newly accumulated charge is partially transferred and read out. As a result, a short accumulation time signal is obtained.
2. In the final sub-frame, the accumulated charge is partially drained and charge accumulation is repeated.
3. Finally, a whole charge transfer operation is done and the transferred signal to the floating diffusion (FD) is read out.

From the operation, two set of output signals is obtained from a single photodiode, the long and short accumulation time signals. A Wide dynamic range image can be synthesized from the two setS of acquired signals because the difference of charge accumulation time can sufficiently expand the dynamic range of the sensor.

The signal from wholly transferred charge in the final sub-frame determines which signals have to be used, whether the long accumulation or the partially transferred short accumulation time signals. A method to judge which signals to be used is proposed. If the quantity of accumulated charge reaches a threshold value, *Q_T_* at the end of final sub-frame and it is read out, the short accumulation signal is selected, if it is less than *Q_T_*, the long accumulation time signal is used. The most important task in operating this sensor is identifying the value of *Q_T_*.

In the case of a weak light irradiated on the pixel, the same operation (1)∼(3) is performed. However, the read data at the end of first sub-frame is 0 because the accumulated charge in photodiode does not exceed threshold level, *Q_T_*. In the final sub-frame, the accumulated charge also does not exceed *Q_T_*. Therefore, only the long accumulation signal is used in synthesized wide dynamic range image prior to the output signal in final sub-frame does not exceed the s threshold value, *Q_T_*.

## 4. Principle of Charge Transfer

The charge transfer mechanism plays an important role in this sensor. Hence, two type of charge transfer namely the whole charge transfer and partial charge transfer mechanism are described in this section.

### 4.1. Whole Charge Transfer

**Figure 2.**
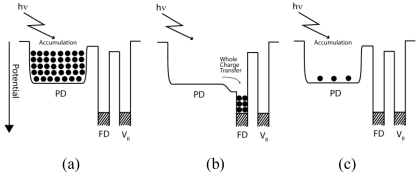
The principle of Whole Charge Transfer, **(a)** charge accumulation **(b)** charge transfer **(c)** new charge accumulation.

The whole charge transfer is the same as a normal charge transfer in conventional 4T APS CMOS image sensors [14]. As shown in Figure 2(a), the signal charge is accumulated in the photodiode starting from the initial state. The accumulation period is one frame for a normal 4T-APS which is equal to two sub-frames in the image sensor proposed in this work. The whole charge transfer operation is done after the accumulation period end and the transferred signal charge is read out subsequently as shown in Figure 2(b). Then the next frame with a new accumulation starts all over again as in Figure 2(c). To obtain a perfect charge transfer, the transfer gate voltage must be able to increase the potential barrier under itself to be higher than the potential of photodiodes within an appropriate time until accumulated charge is perfectly transferred because the potential of photodiode increases as the accumulated charge is transferred to the FD.

The saturated accumulated signals charge can cause smear and blooming [15] in the regenerated image for the conventional four transistors APS. To prevent this problem, some sensors come with a shorter accumulation time, but this will decrease the accumulated signal in low illumination region and results in a lower signal-to-noise ratio (SNR). Therefore, the proposed technique may solve this problems.

### 4.2. Partial Charge Transfer

**Figure 3.**
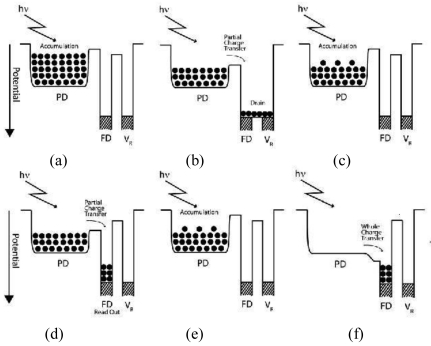
The principle of Partial Charge Transfer.

The partial charge transfer mechanism is described in Figure 3. If in the whole charge transfer all accumulated charges are transferred at once, it is different in the case of the partial charge transfer. As its name suggests, only a part of the accumulated charges are drained, transferred and read out, subsequently.

In Figure 3(a), the signal charge is accumulated in the photodiode starting from the initial state followed by partial charge transfer for draining purpose, Figure 3(b). The charge accumulation process starts once again, Figure 3(c), followed by partial charge transfer for read out, Figure 3(d). Then charge accumulation process start once again, Figure 3(e) and finally the whole charge transfer operation take place in the final sub-frame, Figure 3(f).

To assure partial charge transfer works properly, an appropriate transfer gate voltage must be applied to increase the potential barrier under the transfer gate to be higher than the potential of the photodiode until part of the accumulated charge is transferred. After a short time, the charge transfers stop because the increment in photodiode potential as the accumulated charge is transferred to the FD. As a result, only a part of accumulated charge is transferred. This mechanism is the same in the case of partial charge transfer for draining or signal reading out purposes. The frequency of the partial charge transfer operation for read out purpose as illustrated in Figure 3 is once, followed by the whole charge transfer operation in the final sub-frame.

## 5. Non-Linearity due to Current Diffusion

**Figure 4.**
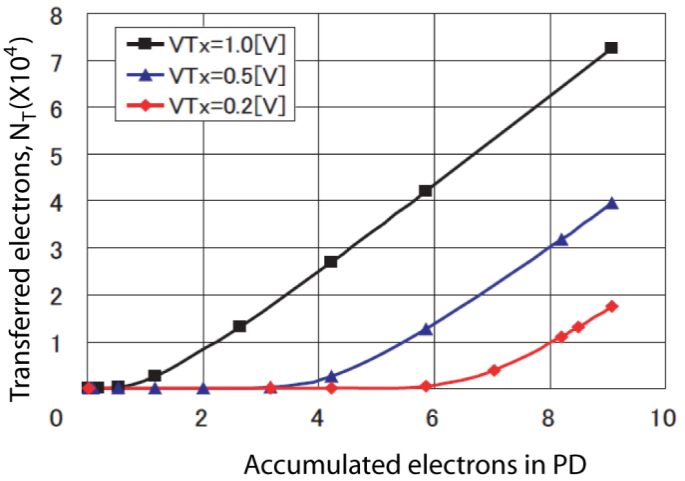
Partially transferred electrons vs accumulated electrons in PD.

A simulation to check the characteristics of partially transferred electrons, *N_T_* with respect to the accumulated electrons in PD and *V_TX_* has been done using the SPECTRA, a simulator built especially for simulating CMOS image sensor's pixel and CCD. The first step for simulation starts with drawing the layout in cadence and the file are transferred to the SPECTRA input file. Then, the parameters are specified and followed by running the SPECTRA in transient mode. In the simulation, the transfer time is set to 0.5 μs. The simulation results are shown in Figure 4. From the figure, it can be said that if the accumulated electrons in PD is greater than threshold value, *Q_T_*, the transferred electrons has a linear response. However, in the region near to the *Q_T_*, it has a non-linear response due to carrier diffusion. The non-linear relation between transferred charge and potential barrier under transfer gate can be calculated by the principle of current diffusion.

**Figure 5.**
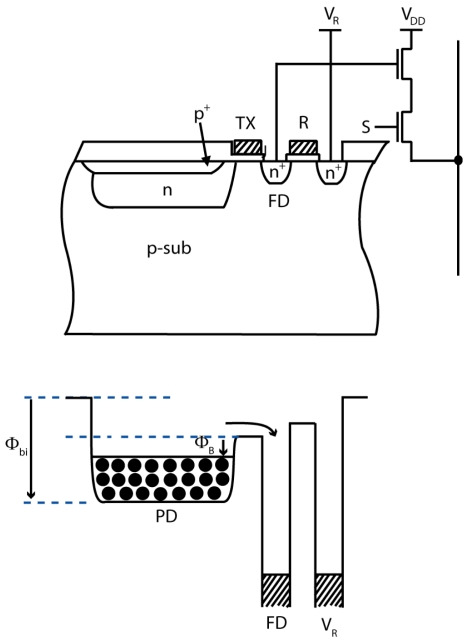
The pixels cross section and potential profile of a photodiode.

Figure 5 shows the pixels cross section and potential profile of a photodiode. The diffusion current or sub-threshold current in a semiconductor is given by:

(1) $$J=-qD_{n}\frac{\delta n_{p}}{\delta x}\cdot A$$

where:

(2) A=W×d

In the equation, *D_n_, n_p_, x, A* and *d* are the diffusion coefficient, minority carrier density, variable of diffusion length at x axis, area and depth of the channel for current flow, respectively. The *D_n_* is given by Einstein's relation:

(3) $$D_{n}=\frac{kT}{q}\mu_{n}$$

δn_p_/δ_x_ in Equation (1) is calculated as:

(4) $$-\frac{\delta n_{p}}{\delta x}=\frac{n_{p}(0)-n_{p}(L)}{L}$$

where, L is the diffusion length and:

(5) $$n_{p}(0)=n_{p0}\exp(\frac{\Phi_{S}}{V_{T}})$$

and:

(6) $$n_{p}(L)=n_{p0}\exp(\frac{\Phi_{S}-V_{D}}{V_{T}})\approx 0$$

Calculating Equations (1)–(6), the diffusion current can be written as:

(7) $$J=\mu_{n}\frac{W}{L}\text{kTd}n_{p0}\exp(\frac{\Phi_{S}}{V_{T}})$$

Note that *Φ_S_* is the surface potential of silicon at transfer gate and can be calculated as:

(8) $$\Phi_{S}=\Phi_{bi}-\Phi_{B}$$

By substituting the Equation (8) into (7), the diffusion current can be rewritten as:

(9) $$J=\mu_{n}\frac{W}{L}\text{kTd}n_{p0}\exp(\frac{\Phi_{bi}-\Phi_{B}}{V_{T}})$$

and equation (9) can thus be simplified to:

(10) $$J=J_{0}\exp(\frac{-\Phi_{B}}{V_{T}})$$

where:

(11) $$J_{0}=\mu_{n}\frac{W}{L}{\text{kTdn}}_{p0}\exp(\frac{\Phi_{bi}}{V_{T}})$$

In the equations:

(12) $$V_{T}=\frac{kT}{q}$$

**Figure 6.**
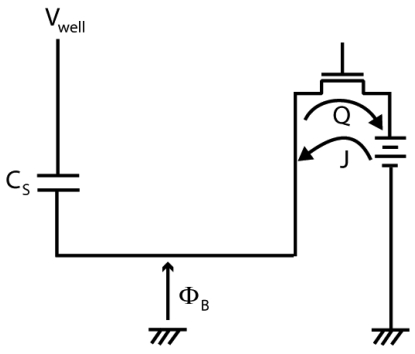
The equivalent circuit.

Next, the equivalent circuit shown in Figure 6 is considered. From the figure, the current flow is given by:

(13) $$J=C_{S}\frac{\delta \Phi_{B}}{\delta t}$$

Differentiation of Equation (10) is:

(14) $$\frac{\delta J}{\delta t}=J_{0}(-\frac{1}{V_{T}})\exp(\frac{-\Phi_{B}}{V_{T}})\cdot \frac{\delta \Phi_{B}}{\delta t}=-\frac{1}{V_{T}}\frac{\delta \Phi_{B}}{\delta t}\cdot J$$

By substituting the Equation (13) into (14), Equation (10) is rewritten as:

(15) $$\frac{\delta J}{\delta t}=-\frac{1}{V_{T}C_{S}}J^{2}$$

Calculate the Equation (15):

(16) $$J=\frac{J(0)}{1+\overset{t}{\;}/\underset{\tau }{\;}}$$

where:

(17) $$\tau =\frac{C_{S}V_{T}}{J_{0}}$$

and *J*(0) is current at t = 0.

From Equation (9), by assuming that *J*(0) = *J*(*Φ*_bi_-*Φ*_B_ = 0), *J*(0) is given by:

(18) $$J(0)=\mu_{n}\frac{W}{L}{\text{kTdn}}_{p0}$$

Using the parameters in Table 2:

(19) $$J(0)=1.31\times 10^{-20}[\text{A}]$$

Within the readout time, the transferred charge is given by:

(20) $$Q_{\text{Trans}}=\int_{o}^{t_{R}}J\delta t=J(0)\tau \ln(1+\overset{t_{R}}{\;}/\underset{\tau }{\;})$$

If 1 ≫ t_R_/τ:

(21) $$Q_{\text{Trans}}=J(0)\tau \overset{t_{R}}{\;}/\underset{\tau }{\;}=J(0)t_{R}$$

If t_R_ = 0.5 [μs], the number of transferred electrons are:

(22) $$N_{\text{Trans}}=J(0)\frac{t_{R}}{q}$$

If one electron is transferred, then, from Equation (18):

(23) $$J_{1}=3.2\times 10^{-13}[\text{A}]$$

At this time, J[0] is renamed as *J*_1_:

From Equations (9) and (18):

(24) $$\frac{J_{1}}{J(0)}=\exp(\frac{\Phi_{bi}-\Phi_{B}}{V_{T}})$$

Substituting Equations (19) and (23) in Equation (24):

(25) $$\Phi_{bi}-\Phi_{B}=0.442\;[\text{V}]$$

and since:

(26) $$\Phi_{bi}=\frac{kT}{q}\ln(\frac{N_{D}N_{A}}{n_{i}^{2}})$$

by substituting the values in Table 2:

(27) $$\Phi_{bi}=0.897\;[\text{V}]$$

Therefore, from Equations (25) and (27):

(28) $$\Phi_{B}=0.455\;[\text{V}]$$

From the above calculations, it is clear that the charge is transferred continuously until the *Φ_B_* reach 0.455 V, then the charge transfer process stops. It also significant that the diffusion current has an exponential relationship with *Φ_B_* which suggests a non-linear relationship between transferred charge and potential barrier under the transfer gate.

## 6. Non-Linearity Due to Initial Condition of a Photodiode and Its Influences on the Dynamic Range Expansion

**Figure 7.**
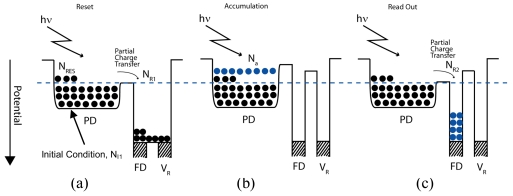
Initial condition influents the read out signals in partial transfer technique.

The initial condition of the photodiode can influence the partially transferred charge for the short accumulation time signal. The Initial condition is indicated by the number of initially accumulated electrons in PD, *N_I1_*. Figure 7 illustrates the partial charge transfer with different initial condition of photodiode. From the figure, when a photodiode with initial condition of 17,000 electrons is partially reset, and the number of drained electron, *N_R1_* is 2,200, some electrons above threshold value *Q_T_, N_RES_* still remains in photodiode. Then, the re-accumulation operation takes place again and if the number of re-accumulated electron *N_a_* is 2,200, the read out short accumulation time signal, *N_R2_* in Figure 7 (c) should be the same as *N_a_*. Therefore, the relation between *N_R1_, N_a_* and *N_R2_* can be conclude as:

(29) $$\text{if}\;N_{R1}=N_{a}\to N_{R2}=N_{a}$$

and:

(30) $$\text{if}\;N_{R1}\neq N_{a}\to N_{R2}\neq N_{a}$$

The relationship between *N_R1_, N_a_* and *N_R2_* of Equation (30) can contribute to the readout error in the short accumulation signal of the sensor.

**Figure 8.**
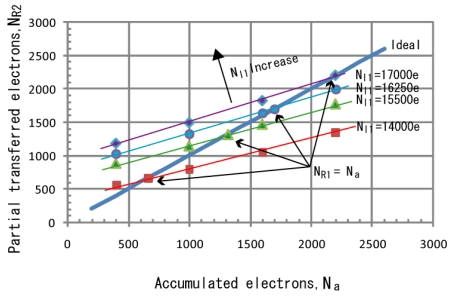
Partial transferred electrons for read out, *N_R2_* versus accumulated electrons within short accumulation time, *N_a_*.

An analysis of the non-linearity due to the partial charge transfer has been conceded. A simulation is carried to check the relations of re-accumulated electrons and partially transferred electrons with respect to the initial conditions of the photodiode. In the simulation, the transfer gate drive voltage and charge transfer time is set to 0.5 V and 1.0 μs, respectively. The simulation results are shown in Figure 8, which shows the relationship between *N_R2_* and *N_a_*, deviates from the ideal curve. As the number of initially accumulated electrons in PD, *N_I1_* is increased, the *N_R2_* - *N_a_* curve moves upward which means the error for low number of accumulated electron is increased.

**Figure 9.**
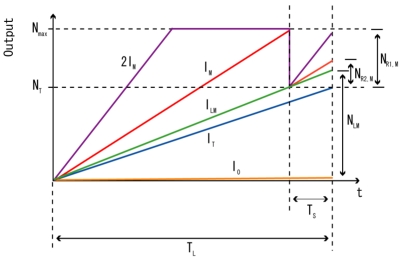
Charge accumulation in one frame.

The simulated relationship between *N_R2_* and *N_a_* which deviates from the ideal curve can contribute to the non-linearity in the synthesized wide dynamic range signal. Figure 9 shows some conditions of incident light, *I_0_, I_T_, I_LM_, I_M_* and *2I_M_* during the accumulation of one frame. From the figure, some equations can be derived as:

(31) $$qN_{T}=I_{T}\cdot T_{L}$$

(32) $$qN_{LM}=I_{LM}\cdot T_{L}$$

(33) $$q(N_{LM}-N_{T})=I_{LM}\cdot T_{S}$$

(34) $$qN_{LM}=I_{LM}\cdot T_{L}$$

where, *T_L_* and *T_S_* are the long and short accumulation time, respectively. Then, the short accumulated signal, *qN_a_*, and the saturation level of accumulated signal, *N_max_* for incident light of *I_M_* can be written as:

(35) $$qN_{a}=I_{M}\cdot T_{S}$$

(36) $$qN_{\max}=I_{M}(T_{L}-T_{S})$$

From Equations (35) and (36), *N_a_* can be calculated as:

(37) $$N_{a}=\frac{T_{S}}{T_{L}-T_{S}}\cdot N_{\max}$$

For example, if *N_max_* is set to 17,000 electrons and the ratio of *T_L_* and *T_S_* is set to 21:1, the *N_a_* is equal to 850 electrons and by referring to the simulation results in Figure 9, the read out electron, *N_R2_*, at *N_a_* equals 850 electrons, is simulated to be 1,400 electrons, different from the ideal value of 850 electrons by almost 65%. A calculation has been done from the simulation data of Figure 8 to study the effects of changing the ratio of *T_L_* to *T_S_*, and the saturation level of the photodiode, *N_max_* to the linearity in short accumulation signal region of wide dynamic range signals. Since, the wide dynamic range signal is synthesized from the two sets of long and short accumulation time signals using the equations:

(38) $$N_{\text{out}}=X_{L}\;\left(if,X_{L}<N_{T}\right)$$

and:

(39) $$N_{\text{out}}=X_{S}\times \frac{T_{L}}{T_{S}}\;\left(if,X_{L}\geq N_{T}\right)$$

the non-linearity only affects the short accumulation signal obtained from partial transferred read out.

**Figure 10.**
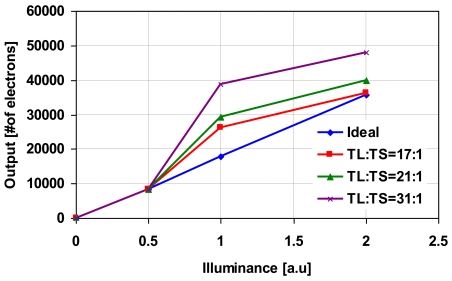
Photo-electric conversion characteristics of the synthesized wide dynamic range signals.

**Figure 11.**
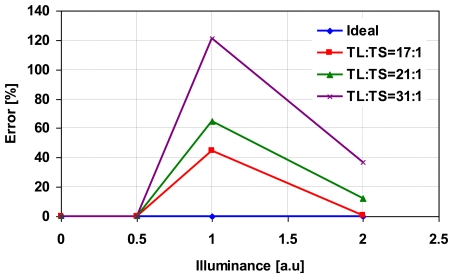
Non-linearity in high illumination region (error in %).

Figure 10 shows the photo-electric conversion characteristics of the synthesized wide dynamic range signals with the parameter of the ratio of *T_L_* to *T_S_* is set to 17:1, 21:1 and 31:1. In the calculation, *N_max_* is set to 17,000 electrons. As the ratio of *T_L_* to *T_S_* is greater, non-linearity at high illumination region becomes worst. The error explained in percentages is shown in Figure 11. The error is increased as the ratio of *T_L_* to *T_S_* is increased because *N_a_* is decreased due to shorter accumulation period and from the simulated results in Figure 8, the error at lower numbers of *N_a_* is higher than the error at high numbers of *N_a_*.

**Figure 12.**
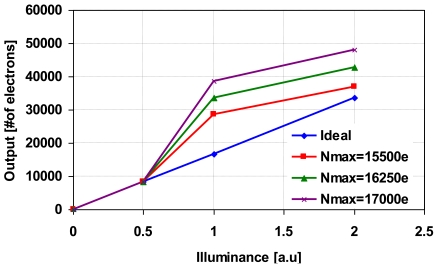
Photo-electric conversion characteristics of the synthesized wide dynamic range signals.

**Figure 13.**
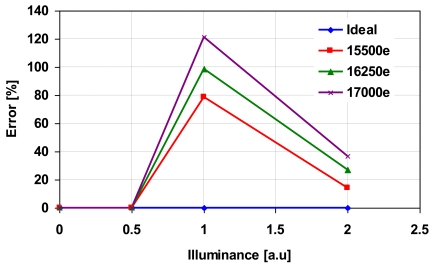
Non-linearity in high illumination region (error in %).

Figure 12 shows the photo-electric conversion characteristics of the synthesized wide dynamic range signals with the parameter of *N_max_* set to 15,500 electrons, 16,250 electrons and 17,000 electrons. In the calculation, the ratio of *T_L_* to *T_S_* is set to 31:1. When *N_max_* is large, non-linearity at high illumination region becomes worst. The error explained in percentages is shown in Figure 13. As *N_max_* increases, the line in Figure 8 moves upwards, therefore despite the same *N_a_*, the error is larger for *N_a_* with higher *N_max_*. From this analysis, it is concluded that the error increases as *N_max_* increases. The dynamic range expansion ratio, *R_DE_*, in this technique is given by:

(40) $$R_{DE}=\frac{T_{L}}{T_{S}}$$

Therefore, the dynamic range in this technique can be expanded either by using a photodiode with higher *N_max_* or by increasing the ratio of *T_L_* to *T_S_*. However, as discussed, the higher *N_max_* and ratio of *T_L_* to *T_S_* contributes to higher error in high illumination region, result in non-linearity in the synthesized wide dynamic range signals. Thus, the optimized *N_max_* and the ratio of *T_L_* to *T_S_* must be considered to reduce this error for obtaining a linear response in the synthesized wide dynamic range signals. Furthermore, solutions such as double mid-point shutter technique [7] may be applicable for reducing this error.

## 7. Conclusions

The partial charge transfer technique is a countermeasure to improve dynamic range of CMOS image sensors and at the same time maintains a high fill factor because only one photodiode is integrated in each pixel. The dynamic range expansion in this sensor is controlled by partial charge transfer and if a very wide dynamic range is required, it can be achieved by taking a large accumulation ratio of the long to the short accumulation time signals. However, the technique suffers from non-linearity in the output of the synthesized wide dynamic range signals especially if a large accumulation ratio is taken. An analysis of the non-linearity utilizing this technique has been done and discussed. The calculation and simulation results show that non-linearity can be caused by two factors that are current diffusion from the potential well and initial conditions of photodiode. From the calculations, it is shown that the diffusion current has an exponential relationship with the potential barrier suggesting a non-linear relationship between the transferred charge and potential barrier under the transfer gate. The simulation results show that the error in the high illumination region is increases as the ratio of the long to the short accumulation time is increases. Furthermore, increasing the saturation level of photodiodes also increases the error in the high illumination region.

## Figures and Tables

**Table 1.** Pixel characteristics.

Technology	0.18 μm CIS 1P4M
Pixel size	7.5 μm × 7.5 μm
No. of photodiode	1
Photodiode shape	Octagonal
Fill factor	14%

**Table 2.** Device parameters.

**Parameter**	**Value**
*W*	1.5 [μm]
*L*	0.7 [μm]
*μ_n_*	700 [cm^2^/V·S]
*d*	0.1 [μm]
*N_D_*	2 × 10^17^ [cm^−3^]
*N_A_*	10^18^ [cm^-3^]
*n_i_*	1.45 × 10^10^ [cm^−3^]
